# Early Glucose Variability Is Associated with Mortality in Critically Ill Children: A Retrospective Pediatric Intensive Care Study

**DOI:** 10.3390/nu18142304

**Published:** 2026-07-14

**Authors:** George Briassoulis, Maria Biliraki, Petros Stathakis, Panagiotis Briassoulis, Stavroula Ilia

**Affiliations:** 1 Postgraduate Program “Emergency and Intensive Care of Children, Adolescents and Young Adults”, School of Medicine, University of Crete, 70013 Heraklion, Greece; mariabiliraki@yahoo.gr (M.B.); stathakispetros96@gmail.com (P.S.); briaspan@med.uoa.gr (P.B.); stavroula.ilia@uoc.gr (S.I.); 2 Second Department of Anaesthesiology, Attikon University Hospital, School of Medicine, National and Kapodistrian University of Athens, 11527 Athens, Greece; 3 Paediatric Intensive Care Unit, University Hospital, School of Medicine, University of Crete, 70013 Heraklion, Greece

**Keywords:** glucose variability, pediatric intensive care, critically ill children, mortality, organ dysfunction, prognostic markers

## Abstract

**Background:** Critical illness is frequently accompanied by dysregulated glucose homeostasis, resulting in increased glucose variability (GV). Beyond absolute hyperglycemia or hypoglycemia, early glycemic instability may reflect disease severity and metabolic stress in critically ill children. **Objective:** To evaluate the prognostic value of early GV indices during the first 72 h of pediatric intensive care unit (PICU) admission and to examine their association with organ dysfunction and adverse outcomes. **Methods:** This retrospective observational study included children admitted to the PICU between October 2022 and July 2024 with a length of stay greater than 72 h. Clinical and laboratory data were extracted from electronic health records. GV indices were calculated from glucose measurements obtained during the first 72 h after admission and included mean absolute glucose change (MAG), glycemic lability index (GLI), standard deviation (SD), coefficient of variation (CV), and average consecutive absolute change percentage (ACACP). Associations with severity of illness and outcome were assessed using correlation analysis, exploratory multivariable logistic regression, and receiver operating characteristic (ROC) analysis. **Results:** A total of 248 patients were included (mean age 7.1 ± 5.9 years; 143 [57.7%] male; mortality 7.3%), with a mean of 15.9 ± 4.0 glucose measurements per patient during the first 72 h. GV indices were strongly intercorrelated (all *p* < 0.001) and were associated with PELOD-2 and/or lactate levels (*p* < 0.05). Non-survivors had higher GV values than survivors. In exploratory multivariable logistic regression, SD (OR 4.82, 95% CI 2.2–10.4, *p* < 0.001) and PELOD-2 score (OR 1.68, 95% CI 1.3–2.2, *p* = 0.004) were associated with mortality. In ROC analysis, SD and PELOD-2 showed similarly strong discrimination for PICU mortality, with no significant difference by DeLong testing. GLI (AUROC 0.70, *p* = 0.004), ACACP (AUROC 0.68, *p* = 0.005), and CV (AUROC 0.66, *p* = 0.027) showed fair discrimination, whereas MAG and lactate were not significant predictors. **Conclusions:** In this single-center retrospective PICU cohort, early glucose variability during the first 72 h of admission was associated with illness severity and PICU mortality. Among the evaluated indices, SD showed the strongest association with mortality and remained associated with outcome together with PELOD-2 in an exploratory model. These findings should be interpreted as associative and hypothesis-generating; prospective multicenter studies with standardized glucose monitoring and formal incremental prediction analyses are required before GV can be incorporated into routine prognostic assessment.

## 1. Introduction

Glucose dysregulation is common during critical illness and reflects the combined effects of neuroendocrine stress, inflammation, insulin resistance, organ dysfunction, nutritional support, and therapeutic interventions. In children admitted to the pediatric intensive care unit (PICU), hyperglycemia, hypoglycemia, and wider fluctuations in blood glucose have all been associated with adverse outcomes [1,2,3,4]. However, isolated admission or daily glucose values provide only a partial view of metabolic instability because they do not capture the dynamic oscillations that occur during the early phase of critical illness [2,5].

Glycemic variability (GV), defined as the degree of fluctuation in blood glucose values over time, has emerged as an additional component of dysglycemia beyond hyperglycemia and hypoglycemia [5,6]. This concept is clinically relevant because rapid glucose oscillations may induce more oxidative stress and endothelial dysfunction than sustained hyperglycemia, and may contribute to inflammatory activation, mitochondrial injury, microvascular dysfunction, and organ injury [5,6,7,8]. These mechanisms provide a biologically plausible link between GV and adverse outcomes in critically ill patients, including those with sepsis, neurologic injury, cardiovascular disease, and multiorgan dysfunction [8,9,10,11,12,13].

The prognostic relevance of GV has been extensively investigated in adult critical illness. In sepsis, early studies showed that glucose variability was independently associated with mortality and that indices incorporating glycemic lability could outperform mean glucose values [8,14]. More recent adult ICU studies and a systematic review/meta-analysis in sepsis have confirmed that elevated GV is associated with higher mortality, although the strength of association differs according to the GV metric used, the presence of diabetes, illness severity, and the method and timing of glucose measurement [10,15,16,17,18]. These observations suggest that GV may be a marker of acute metabolic instability rather than simply a reflection of mean glucose concentration [10,11,12].

Several indices have been proposed to quantify GV. Standard deviation (SD) and coefficient of variation (CV) are simple and widely used dispersion-based measures, with CV adjusting SD for mean glucose concentration [5,6]. Other indices, such as mean absolute glucose change (MAG) and glycemic lability index (GLI), incorporate temporal information by considering the magnitude and rate of change between consecutive glucose measurements [14,19]. Average consecutive absolute change percentage (ACACP) has also been proposed as a practical metric reflecting relative changes between consecutive glucose values [19]. Because these indices capture partly different aspects of glycemic instability, they should not be considered interchangeable in clinical or prognostic studies [5,6,19,20]. Accordingly, SD mainly reflects overall dispersion around the individual mean, CV expresses dispersion relative to mean glucose, MAG and GLI emphasize the magnitude and timing of consecutive changes, and ACACP reflects relative percentage changes between adjacent measurements.

Evidence in critically ill children remains more limited than in adults. In early pediatric PICU research, glucose variability was associated with survival, raising the question of whether GV represents adaptive allostasis, harmful instability, or both [2]. Subsequent pediatric studies suggested that GV is independently associated with mortality, but the best-performing index has differed across cohorts. Du et al. found GLI to be independently associated with mortality in critically ill children, whereas Dong et al., in a multicenter pediatric PICU cohort, found MAG to have the strongest predictive performance among several GV indices calculated during the first 72 h of PICU stay [3,4]. These differences highlight the need for further pediatric studies comparing multiple GV indices in clinically relevant early time windows [3,4]. The novelty of the present study is not the evaluation of GV per se, but the simultaneous comparison of five GV indices derived from routine central laboratory arterial glucose measurements during the early PICU course in a non-diabetic pediatric cohort, while also examining their relationship with PELOD-2, lactate, fluid administration, enteral nutrition, and PICU mortality. This approach was intended to reflect real-world intermittent glucose monitoring rather than continuous glucose monitoring or protocolized research sampling.

The first 72 h after PICU admission represent a critical period during which systemic stress, hemodynamic instability, organ dysfunction, vasoactive support, nutritional changes, and treatment escalation are often most pronounced [19,21]. Accordingly, the 72 h window was selected because it corresponds to the early phase used in previous pediatric GV studies and captures the period during which acute stress responses, vasoactive support, organ dysfunction, fluid and nutritional changes, and treatment escalation are most likely to influence glucose dynamics. This window also provides sufficient serial measurements for calculating time-dependent GV indices while remaining close enough to admission to preserve potential prognostic relevance. We therefore aimed to evaluate five GV indices calculated from serial central laboratory arterial blood glucose measurements obtained during the first 72 h of PICU stay in critically ill children without diabetes. We examined their relationships with glucose profiles, lactate, illness severity, fluid and nutrition variables, and PICU mortality, and compared their prognostic performance with PELOD-2. 

## 2. Materials and Methods

### 2.1. Study Design and Setting

This was a retrospective observational study conducted in a tertiary pediatric intensive care unit. Consecutive critically ill children admitted to the PICU between October 2022 and July 2024 were screened for eligibility. The study focused on glucose measurements obtained during the first 72 h after PICU admission and evaluated the association of glucose variability indices with clinical characteristics, laboratory variables, fluid administration, enteral nutrition, illness severity, and PICU mortality.

### 2.2. Study Population

Children younger than 18 years of age admitted to the PICU with a length of stay longer than 72 h were eligible for inclusion. Patients were included if serial arterial blood glucose measurements were available from the central laboratory during the first 72 h of PICU stay, with sufficient frequency to allow calculation of glucose variability indices. Patients with diabetes mellitus, known endocrinopathies, treatment with insulin or systemic corticosteroids during the observation period, incomplete glucose data, or missing outcome data were excluded.

The exclusion of patients receiving insulin or systemic corticosteroids was intended to reduce major treatment-related confounding of glucose variability and to focus on stress-related glycemic instability. We acknowledge, however, that this approach may have excluded some of the sickest patients and may limit external validity to broader PICU populations in which insulin or corticosteroids are commonly used.

### 2.3. Data Collection

Data were extracted from electronic medical records. Demographic and clinical variables included age, sex, body mass index, BMI z-score, admission type, medical or surgical admission, diagnostic category, presence of infection at admission, comorbidities, use of mechanical ventilation, vasoactive drug support, duration of mechanical ventilation, PICU length of stay, and survival status. Illness severity was assessed using the Pediatric Logistic Organ Dysfunction-2 score (PELOD-2).

Laboratory variables included arterial blood glucose concentrations and lactate levels recorded during the first 72 h of PICU stay. Fluid- and nutrition-related variables included the type and volume of intravenous fluids administered, daily fluid balance, and daily enteral caloric intake during the same 72 h period.

Diagnostic categories included infection, respiratory failure, surgical conditions, severe trauma, neurological disorders, and other admission diagnoses.

### 2.4. Blood Glucose Measurements

Central laboratory arterial blood glucose values recorded during the first 72 h after PICU admission were analyzed. For each patient, the total number of glucose measurements obtained during the first 72 h was recorded. Daily mean, maximum, and minimum glucose concentrations were calculated separately for day 1, day 2, and day 3. Glucose concentrations are presented in mg/dL in the descriptive analyses. When required for glucose variability calculations, glucose values were converted between mg/dL and mmol/L according to the requirements of EasyGV software, version 10 (Oxford University Innovation, Oxford, UK), or the corresponding published formula.

Glucose measurements were obtained as part of routine PICU care and were not performed according to a study-specific protocol. In mechanically ventilated or hemodynamically unstable patients, arterial blood gas and laboratory monitoring were typically repeated according to clinical status, treatment changes, and physician judgment. Therefore, the timing and frequency of glucose measurements were clinically driven rather than standardized. During the study period, the unit did not use continuous glucose monitoring for routine care. Hyperglycemia was managed conservatively, with confirmation of abnormal values, review of glucose-containing fluids and nutritional intake, avoidance of excessive glucose administration, and individualized insulin therapy only when clinically indicated; patients who received insulin during the observation period were excluded from the present analysis. Hypoglycemia was treated promptly according to standard PICU practice with intravenous glucose administration and adjustment of nutritional or fluid prescription. Triglyceride measurements were not systematically obtained in all patients during the first 72 h and therefore were not included in the primary analysis.

### 2.5. Glucose Variability Indices

Glucose variability was assessed using five indices calculated from serial glucose measurements obtained during the first 72 h of PICU stay: standard deviation (SD), coefficient of variation (CV), mean absolute glucose change (MAG), glycemic lability index (GLI), and average consecutive absolute change percentage (ACACP). SD is a widely used and relatively robust measure of glucose dispersion [22,23], while CV represents SD corrected for mean glucose concentration [24]. MAG [25] and GLI [26] incorporate temporal information by considering the magnitude and rate of change between consecutive glucose measurements and the time interval between them. ACACP represents the mean percentage change between consecutive glucose measurements and has been proposed as a practical index that may support real-time clinical assessment of glycemic instability [27].

SD, CV, and MAG were calculated using EasyGV software, version 10 (Oxford University Innovation, Oxford, UK), a validated Excel-based tool for the automated assessment of glucose variability metrics. GLI and ACACP were calculated separately according to previously published formulas. All indices were calculated using the same dataset of serial glucose measurements obtained during the first 72 h.

MAG was calculated as the sum of absolute differences between consecutive glucose values divided by the total observation time in hours:

$$MAG=\frac{\sum_{n=1}^{N-1}\left|G_{n}-G_{n+1}\right|}{T}$$

where *G_n_* and *G_n+_*_1_ represent consecutive glucose measurements, *N* is the total number of glucose measurements, and *T* is the total observation time in hours.

GLI was calculated from the squared differences between consecutive glucose values divided by the corresponding time interval between measurements:

$$GLI=\sum_{n=1}^{N-1}\frac{{\left(G_{n}-G_{n+1}\right)}^{2}}{t_{n+1}-t_{n}}$$

where *G_n_* and *G_n_*_+1_ represent consecutive glucose measurements, and *t_n_* and *t_n_*_+1_ represent the corresponding time points.

SD was calculated as the dispersion of glucose values around the individual mean glucose concentration:

$$SD=\sqrt{\frac{\sum_{i=1}^{n}{\left(X_{i}-\bar{X}\right)}^{2}}{n-1}}$$

where *X_i_* represents each glucose value, *bar*{*X*} the mean glucose concentration, and *n* the total number of glucose measurements.

CV was calculated as SD divided by the mean glucose concentration:

$$CV=\frac{SD}{\bar{X}}$$

ACACP was calculated as the mean percentage of absolute consecutive glucose changes:

$$ACACP=\frac{1}{n_{i}-1}\times \sum \left|\frac{X_{i}-X_{i-1}}{X_{i}}\right|\times 100$$

where *X_i_* represents each glucose value and *X_i_*_−1_ the preceding glucose value.

### 2.6. Intravenous Fluids and Enteral Nutrition

Intravenous fluid administration was assessed during each of the first 3 days of PICU stay. For each 24 h period, the total volume of intravenous fluids administered and the daily intravenous fluid balance were recorded. Intravenous fluids were classified according to type, including balanced crystalloids, such as Plasma-Lyte and Ringer’s lactate, and other administered solutions.

Enteral nutrition was assessed by recording the total daily caloric intake during the first 3 days of PICU stay. Daily enteral caloric intake was compared between survivors and non-survivors and examined in relation to glucose variability indices.

### 2.7. Outcome

The primary outcome was PICU mortality. Patients were classified as survivors or non-survivors according to survival status at PICU discharge. Secondary analyses explored associations between glucose variability indices and illness severity, as reflected by PELOD-2, as well as PICU length of stay and duration of mechanical ventilation.

### 2.8. Ethical Approval

The study was approved by the Administrative Board, the Scientific Council of the University General Hospital of Heraklion, and the Bioethics Committee (Approval No. 14403, Date: 23 September 2022). Permission to access anonymized clinical data was also obtained from the PICU director. Because of the retrospective observational design of the study and the use of anonymized data, the requirement for written informed consent was waived.

No physical or electronic patient documents were removed from the medical records. Data were collected anonymously, although a unique registry number was retained for each patient to allow data verification and matching during the study process.

### 2.9. Statistical Analysis

Data completeness was assessed before analysis. Patients with missing outcome data or insufficient glucose measurements to calculate GV indices were excluded. For the variables included in the primary analysis, no imputation was performed. Analyses were conducted using available complete data for each variable. Fluid balance and enteral nutrition variables were available from daily PICU charts for the first 72 h. Laboratory variables not systematically measured in all patients, such as triglycerides, were not included in the primary analysis. Missingness was less than 5% for all analyzed variables: pH and HCO3 at admission had 3 missing values, type of fluids on day 1 had 5 missing values, and GLI had 10 missing values because of incomplete timing data between consecutive glucose measurements. There were no missing values for fluid quantity, enteral or parenteral nutrition, clinical data, demographic data, outcome, PELOD-2, or the remaining GV indices. No imputation was performed.

Continuous variables were assessed for distribution and are presented as mean ± standard deviation or median and interquartile range, as appropriate. Categorical variables are presented as numbers and percentages. Comparisons between survivors and non-survivors were performed using the independent-samples *t*-test or the Mann–Whitney U test for continuous variables, and the chi-square test or Fisher’s exact test for categorical variables, as appropriate.

Temporal changes in glucose measurements, intravenous fluid administration, fluid balance, and enteral caloric intake during the first 3 days of PICU stay were assessed using the Friedman test for related samples. Correlations between glucose variability indices and clinical or laboratory variables were assessed using Pearson or Spearman correlation coefficients, as appropriate. Because this was a retrospective study including all eligible consecutive patients during the predefined study period, no prospective sample-size calculation was performed. The available sample size was therefore determined by the number of eligible PICU admissions with sufficient glucose measurements and complete outcome data. Statistical modeling was interpreted in light of the limited number of mortality events.

Multivariable logistic regression was used as an exploratory analysis to identify variables associated with PICU mortality. Because only 18 deaths occurred, model construction was intentionally parsimonious to reduce overfitting. Candidate variables were selected on the basis of clinical relevance, univariable association with mortality, and avoidance of redundancy. Given the strong intercorrelations among GV indices, all five GV metrics were not forced simultaneously into the same final model. Collinearity was assessed using variance inflation factors (VIFs), and variables with substantial collinearity were not retained together. The final parsimonious model included SD and PELOD-2, selected on the basis of clinical relevance, univariable association with mortality, and avoidance of collinearity. Results are reported as odds ratios (ORs) with 95% confidence intervals (CIs). Model discrimination was assessed using ROC analysis, while calibration was assessed using the Hosmer–Lemeshow goodness-of-fit test and the Brier score. Because of the small number of events, the multivariable results should be interpreted as exploratory and hypothesis-generating. Because multiple exploratory comparisons and correlations were performed, results were interpreted according to effect size, consistency, and biological plausibility rather than *p*-values alone. No formal correction for multiplicity was applied, and this is acknowledged as a limitation.

Receiver operating characteristic curve analysis was performed to evaluate the discriminative ability of glucose variability indices and PELOD-2 for PICU mortality prediction. Areas under the ROC curve were calculated with 95% confidence intervals. ROC analyses were performed using available complete data for each variable. Pairwise DeLong comparison between SD and PELOD-2 was performed using cases with complete paired data for both variables. Optimal cut-off values were identified using Youden’s index. A two-sided *p*-value < 0.05 was considered statistically significant. Statistical analyses were performed using IBM SPSS Statistics, version 31 (IBM Corp., Armonk, NY, USA).

## 3. Results

### 3.1. Patient Characteristics

A total of 248 patients were included in the study. The mean age was 7.1 ± 5.9 years, and 143 patients (57.7%) were male. Glucose measurements were frequent during the first 72 h after PICU admission, with a mean of 15.9 ± 4.0 measurements per patient. Most admissions were medical rather than surgical (79.6%) and emergency rather than elective (97.2%). The most common diagnostic categories were infection (*n* = 97, 39.1%), respiratory failure (*n* = 46, 18.5%), surgical conditions (*n* = 42, 16.9%), severe trauma (*n* = 35, 14.1%), and neurological disorders (*n* = 11, 4.4%). Baseline demographic and clinical characteristics are presented in Table 1.

### 3.2. Intravenous Fluids and Enteral Nutrition

Total daily fluid volumes, daily intravenous fluid balance, and enteral caloric intake during the first 3 days of PICU stay did not differ significantly between survivors and non-survivors. Across the overall cohort, total intravenous fluid administration increased during the second 24 h period and decreased during the third 24 h period, while fluid balance was significantly lower on day 3 compared with days 1 and 2. In contrast, enteral caloric intake increased progressively during days 2 and 3 compared with day 1 (Supplementary Table S1).

### 3.3. Types of Intravenous Fluids

Balanced crystalloids, mainly Plasma-Lyte or Ringer’s lactate, were the most frequently administered intravenous fluids during the first 3 days. The distribution of fluid types did not differ according to admission characteristics, infection status, comorbidity, mechanical ventilation, diagnostic category, or survival status. Glucose variability indices also did not differ across groups receiving different types of intravenous fluids (Supplementary Figure S1).

### 3.4. Admission Laboratory Findings

Admission laboratory findings are presented in Supplementary Table S2. Non-survivors had higher PaCO_2_ values than survivors, whereas no other admission laboratory variable differed significantly between groups.

### 3.5. Blood Glucose Measurements

Blood glucose measurements were frequent during the PICU stay, with a mean of 15.9 ± 4.0 measurements per patient over the first 72 h. The number of glucose measurements during the first 72 h did not differ significantly between survivors and non-survivors (15.8 ± 4.1 vs. 17.5 ± 3.3, *p* = 0.186), although measurements were numerically more frequent among non-survivors. Mean glucose concentrations did not differ significantly between survivors and non-survivors on days 1–3. Over time, mean glucose values on days 2 and 3 were significantly lower in the overall cohort (*p* < 0.02) and among survivors (*p* < 0.001), whereas no significant temporal change was observed among non-survivors (Table 2).

Maximum glucose concentrations did not differ significantly between survivors and non-survivors on days 1–3, although values were numerically higher among non-survivors on day 1 (149 ± 57 vs. 132 ± 48 mg/dL). Over time, maximum glucose values on days 2 and 3 decreased significantly in the overall cohort (*p* < 0.001) and among survivors (*p* < 0.03), but not among non-survivors (Table 2).

Minimum glucose concentrations also did not differ significantly between survivors and non-survivors on days 1–3. Longitudinally, minimum glucose values on days 2 and 3 decreased significantly in the overall cohort (*p* < 0.001) and among survivors (*p* < 0.001), whereas no significant change was observed among non-survivors (Table 2).

### 3.6. Glucose Variability Indices and Outcome

**Figure 1:**
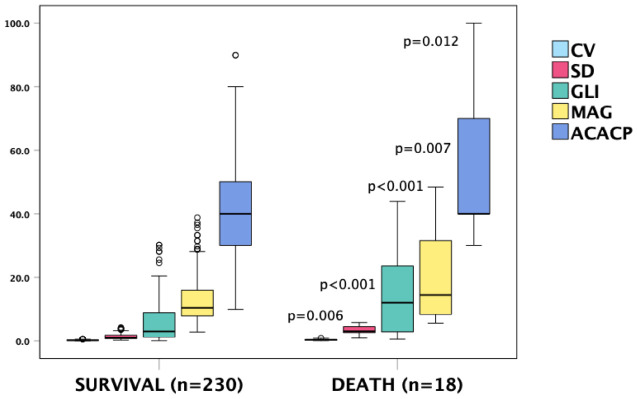
Comparison of glucose variability indices between survivors (*n* = 230) and non-survivors (*n* = 18) based on glucose measurements obtained during the first 72 h of PICU stay. Horizontal lines represent median values, and boxes indicate the interquartile range. Open circles and asterisks indicate outliers.

All five GV indices were significantly higher in non-survivors than in survivors, including MAG, CV, SD, GLI, and ACACP (Figure 1, Table 3). The small non-survivor subgroup should be considered when interpreting all between-group comparisons.

Glucose variability indices did not differ significantly according to BMI z-score, sex, comorbidity status, type of admission, or other demographic and clinical characteristics. 

### 3.7. Interrelationships Among Glucose Variability Indices

**Figure 2:**
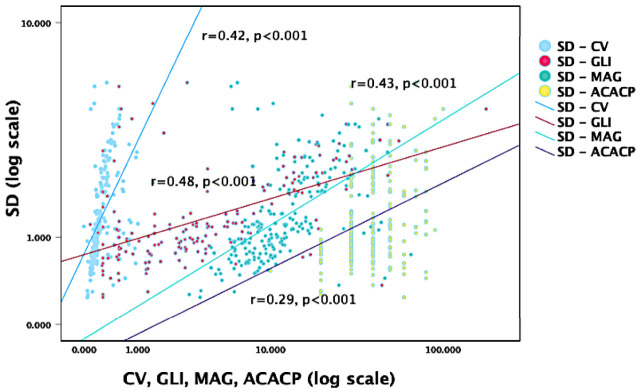
Correlations between SD and the other glucose variability indices, including CV, MAG, GLI, and ACACP. Variables are displayed on logarithmic scales.

The glucose variability indices were strongly intercorrelated, with all pairwise associations reaching statistical significance (all *p* < 0.001). These findings indicate that the indices capture overlapping dimensions of glycemic instability (Figure 2). However, differences in the strength of these correlations suggest that they are not fully interchangeable and may reflect partly distinct aspects of glucose variability.

### 3.8. Correlations with Clinical and Laboratory Parameters

MAG and CV showed the strongest and most consistent correlations with maximum glucose concentrations and weaker correlations with mean glucose values. ACACP and SD were more closely related to mean glucose concentrations, whereas GLI showed weaker and less consistent correlations with daily glucose measurements, particularly on days 2 and 3. Correlations with minimum glucose values were generally weak and inconsistent. Lactate on day 1 correlated weakly with MAG and CV, while all GV indices were significantly associated with PELOD-2, although the magnitude of these correlations was weak to moderate (Table 4).

GV indices were not consistently associated with fluid type, caloric intake, or other clinical and laboratory variables, except for weak correlations between MAG and day-3 fluid balance and between ACACP and day-3 total caloric intake.

### 3.9. Independent Associations of Glucose Variability Indices

In exploratory multivariable logistic regression, model construction was restricted because of the small number of mortality events (*n* = 18). Candidate variables considered for multivariable modeling included clinically relevant variables and variables associated with mortality in univariable analysis. Because of the limited number of events, only two variables were retained in the final model, corresponding to an events-per-variable ratio of 9.0. The GV indices were strongly intercorrelated; therefore, they were not all retained simultaneously in the final model. Collinearity diagnostics did not indicate problematic collinearity between SD (VIF = 1.62) and PELOD-2 (VIF = 1.27) in the final model. In the parsimonious final model, SD and PELOD-2 remained associated with PICU mortality. Higher SD was associated with increased odds of death (OR 4.82, 95% CI 2.2–10.4, *p* < 0.001), while PELOD-2 was also associated with mortality (OR 1.68, 95% CI 1.3–2.2, *p* = 0.004). The model was statistically significant compared with the null model (omnibus χ^2^(2) = 71.93, *p* < 0.001), with Cox and Snell R^2^ = 0.345 and Nagelkerke R^2^ = 0.743. Calibration assessment showed the Hosmer–Lemeshow test = 6.158, *p* = 0.629; Brier score = 0.027. Because of the limited number of events, these estimates should be interpreted cautiously.

### 3.10. ROC Curve Analysis for Mortality Prediction

Receiver operating characteristic (ROC) curve analysis was performed to evaluate the ability of glucose variability indices to discriminate between survivors and non-survivors. Areas under the ROC curve were calculated with 95% confidence intervals. ROC analyses were performed using available complete data for each variable. GLI was available in 238 patients, whereas SD, CV, MAG, ACACP, PELOD-2, and mortality were available in all 248 patients. Among the individual glucose variability indices, SD showed the highest discriminative performance for mortality, with an AUC of 0.877 (95% CI 0.786–0.968, *p* < 0.001). PELOD-2 showed a similarly high AUC (0.875, 95% CI 0.785–0.965, *p* < 0.001) (Table 5). The apparent C-statistic of the final combined exploratory model was AUC 0.949 (95% CI 0.898–0.999, *p* < 0.001).

**Figure 3:**
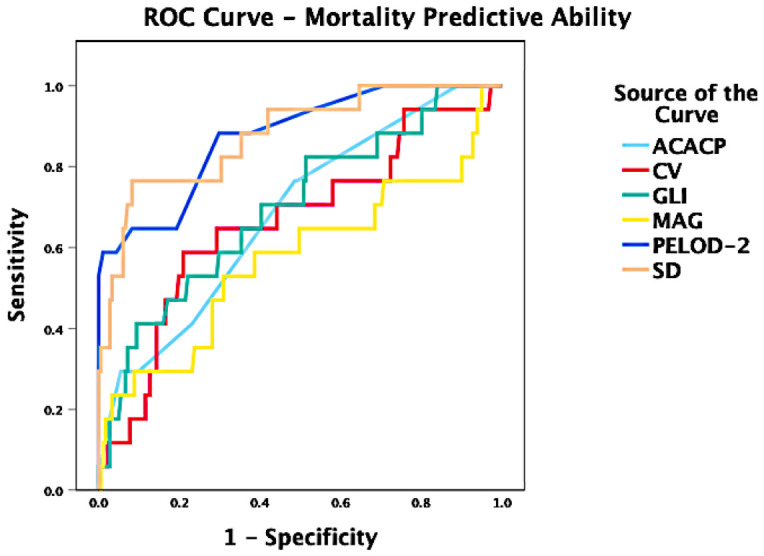
Receiver operating characteristic curves of GV indices and PELOD-2 for PICU mortality prediction. AUC values with 95% CIs are reported in Table 5. SD showed the highest AUC among GV indices, while paired DeLong comparisons showed no significant difference between SD and PELOD-2, shown as the reference severity score.

Among the remaining glucose variability indices, GLI (AUC 0.698, 95% CI 0.563–0.833, *p* = 0.004), ACACP (AUC 0.684, 95% CI 0.555–0.814, *p* = 0.005), and CV (AUC 0.664, 95% CI 0.519–0.809, *p* = 0.027) showed statistically significant but weaker discriminative ability (Figure 3). In contrast, MAG did not significantly discriminate mortality outcome (AUC 0.573, 95% CI 0.405–0.742, *p* = 0.393).

Overall, SD showed the highest AUC among GV indices for mortality discrimination (AUC 0.877, 95% CI 0.786–0.968, *p* < 0.001). PELOD-2 showed a similarly high descriptive AUC (0.875, 95% CI 0.785–0.965, *p* < 0.001) (Supplementary ROC coordinate analysis). 

A pairwise comparison of the correlated ROC curves was conducted using DeLong’s non-parametric test in patients with complete paired SD and PELOD-2 data. In this paired analysis, PELOD-2 and SD showed identical AUCs (PELOD-2 AUC 0.882, 95% CI 0.798–0.967; SD AUC 0.882, 95% CI 0.796–0.968). The AUC difference was negligible and not statistically significant (ΔAUC = 0.002; z = 0.006, *p* = 0.995; 95% CI −0.118 to 0.119), indicating no statistically significant difference in discriminative performance between SD and PELOD-2 (Table 6).

## 4. Discussion

In this single-center retrospective PICU cohort, early GV during the first 72 h of admission was associated with PICU mortality. All five GV indices were higher in non-survivors, but their discriminatory performance and multivariable associations differed. SD showed the strongest association with mortality in this dataset and remained associated with outcome together with PELOD-2 in an exploratory parsimonious model. These findings should not be interpreted as evidence that GV causes mortality or that GV assessment improves established prognostic models. Rather, early GV appears to be a marker of acute metabolic instability and illness severity in this selected population of critically ill children without diabetes, insulin therapy, or systemic corticosteroid exposure.

These findings support the concept that early GV reflects clinically relevant metabolic instability in critically ill children. Unlike isolated hyperglycemia or hypoglycemia, GV captures the dynamic behavior of glucose homeostasis over time [5,6]. This may be particularly relevant in the early PICU course, when counter-regulatory hormone release, systemic inflammation, insulin resistance, vasoactive support, evolving organ dysfunction, nutrition, and therapeutic interventions interact [7,10,12,21]. The significant correlations between all GV indices and PELOD-2 in our cohort suggest that GV is closely linked to illness severity. However, the association between SD and mortality after adjustment for PELOD-2 in the exploratory model suggests that early glucose dispersion may reflect prognostic information not fully captured by conventional severity scoring [1,3,4]. The relationship between GV and PELOD-2 requires cautious interpretation. PELOD-2 reflects the severity of organ dysfunction, whereas GV may reflect the metabolic consequences of systemic stress, inflammation, catecholamine exposure, nutrition, and evolving organ dysfunction. Therefore, the association between SD and mortality after adjustment for PELOD-2 does not necessarily indicate an independent causal pathway. GV may represent either residual severity not fully captured by PELOD-2 or a parallel marker of physiologic instability. Formal incremental prediction analyses and external validation are required to determine whether GV adds clinically useful information beyond established severity scores.

Our results are broadly consistent with the critical care literature, but they also highlight important differences between populations and GV metrics. In the recent sepsis systematic review and meta-analysis, including more than 18,000 patients, high GV was associated with nearly twice the risk of mortality, while continuous analyses showed significant associations for CV, SD, GLI, and MAGE [15]. That meta-analysis suggested that GLI and MAGE may be more reliable prognostic metrics than CV or SD in adult sepsis. In contrast, SD performed best in our pediatric cohort. This discrepancy is not unexpected, because GV indices are influenced by age, diabetes status, severity of illness, glucose sampling frequency, observation window, and whether glucose data derive from continuous glucose monitoring, bedside testing, blood gas analysis, or central laboratory measurements [5,6,15,20].

Comparison with pediatric studies further illustrates this heterogeneity. Rake et al. first raised the clinical relevance of GV in critically ill children, while Du et al. later identified GLI as an independent predictor of mortality in a pediatric PICU cohort [1,3]. Dong et al., in a larger multicenter pediatric PICU study, found MAG to have the highest predictive value among SD, GLI, MAG, and ACACP during the first 72 h of admission [4]. In contrast, our study identified SD as the strongest GV index. Several factors may explain these differences. First, our cohort used central laboratory arterial glucose measurements obtained during routine care, providing consistency of glucose source but not continuous measurement. Second, the timing of glucose sampling was clinically driven and therefore irregular. Third, SD may be relatively robust in datasets with frequent but non-standardized measurements, whereas MAG and GLI may be more sensitive to measurement intervals and may perform better when sampling is standardized or continuous [5,6,19,20]. Fourth, by excluding children with diabetes, endocrinopathies, insulin therapy, or systemic corticosteroid exposure during the observation period, our study focused on stress-related glycemic instability rather than chronic or treatment-driven dysglycemia [16,18].

The stronger performance of SD in this study should therefore be interpreted pragmatically rather than mechanistically. SD is simple, reproducible, easily calculated, and widely understood, but it does not account for the timing or sequence of glucose measurements [5,6]. CV adjusts dispersion for mean glucose and may improve comparability across patients with different average glycemia [5,6]. MAG and GLI incorporate the magnitude and rate of glucose change, which may be advantageous in datasets with standardized or high-frequency glucose monitoring [14,19]. ACACP reflects relative consecutive changes and may be clinically intuitive, but its use in PICU research remains limited [19]. Thus, our findings do not imply that SD is universally superior. Rather, they suggest that in real-world pediatric PICU datasets based on frequent but irregular central laboratory measurements, SD may be a practical and informative marker of early glycemic instability [5,6,19,20].

The absence of strong associations between GV and fluid type, total intravenous fluid volume, fluid balance, or enteral caloric intake is also clinically relevant. In adult ICU studies, GV has sometimes been associated with energy delivery, insulin exposure, and metabolic treatment intensity [28]. In our pediatric cohort, however, GV appeared more closely related to illness severity than to measured fluid administration or enteral caloric intake. This suggests that early GV may primarily reflect the severity of systemic stress, inflammation, organ dysfunction, and metabolic dysregulation rather than simply the amount or type of fluids and calories administered. This interpretation is also consistent with recent sepsis studies linking GV with inflammatory and catabolic phenotypes, acute kidney injury, and combined physiologic variability markers [16,17,20,21].

Recent adult critical care studies increasingly conceptualize GV as part of a broader dynamic risk phenotype rather than as an isolated glucose metric. Combined assessment of GV with stress hyperglycemia ratio, systolic blood pressure variability, or machine-learning models has improved prognostic performance in several adult ICU cohorts, including sepsis, sepsis-associated ARDS, cardiovascular disease, atrial fibrillation, and sepsis-associated acute kidney injury [17,20,21,29,30,31]. Although these approaches are not yet ready for direct translation into pediatric critical care, they support the idea that dynamic physiologic variability may carry clinically meaningful information beyond single static values. Nevertheless, the clinical use of GV should remain interpretative and risk-stratifying rather than treatment-directive.

Importantly, our findings should not be interpreted as support for aggressive glucose lowering or tight glycemic control. Large ICU trials have shown that intensive glucose control may increase hypoglycemia without improving outcomes, and rapid correction or overtreatment may itself increase glucose fluctuations [8]. Continuous glucose monitoring studies in acute illness have also shown that intermittent measurements may miss clinically relevant glucose excursions, particularly hypoglycemia [7]. The clinical implication of our study is therefore not that glucose should be normalized aggressively, but that large early fluctuations may identify children at higher risk who may benefit from closer metabolic monitoring, avoidance of both hyperglycemia and hypoglycemia, and careful integration of glucose trends with illness severity and organ dysfunction.

This study has several limitations. First, its retrospective single-center design precludes causal inference and limits generalizability. Second, only 18 deaths occurred, which limited the number of variables that could be reliably included in multivariable models and increased the risk of overfitting and unstable regression estimates. The multivariable model should therefore be interpreted as exploratory and hypothesis-generating. Third, glucose measurements were obtained during routine care rather than by a standardized protocol or continuous glucose monitoring. Although measurement frequency did not differ significantly between survivors and non-survivors, sampling was clinically driven and may have introduced surveillance bias, particularly for time-dependent indices such as MAG and GLI. Fourth, multiple comparisons, correlations, and ROC analyses were performed without formal correction for multiplicity, increasing the possibility of false-positive findings. Fifth, patients with diabetes, endocrinopathies, insulin therapy, or systemic corticosteroid exposure were excluded to reduce treatment-related confounding, but this may have excluded some severely ill children and limited external validity. Sixth, calibration and incremental prediction analyses were limited, and no external validation cohort was available. Finally, intermittent central laboratory glucose measurements may have missed short-lived hyperglycemic or hypoglycemic excursions detectable by continuous glucose monitoring.

Despite these limitations, the study has several strengths. It focused on a clearly defined non-diabetic pediatric PICU population and reduced major treatment-related confounding by excluding children who received insulin or systemic corticosteroids during the observation period. It used a clinically relevant early 72 h window, analyzed central laboratory arterial glucose measurements, compared five GV indices rather than relying on a single metric, and assessed GV in relation to PELOD-2, lactate, fluid administration, enteral nutrition, and PICU mortality. These features allow a clinically grounded evaluation of whether early GV is associated with outcome and established severity markers in routine pediatric critical care. Future multicenter pediatric studies should use standardized glucose sampling or continuous glucose monitoring, validate the optimal GV metric for children, and determine whether GV-guided monitoring strategies can improve risk stratification without promoting unsafe glucose-lowering practices.

From a practical perspective, the present findings do not support aggressive glucose lowering or tight glycemic control. Instead, marked early GV should be interpreted as a warning signal of metabolic and physiologic instability. Practical next steps include a closer review of glucose trends, avoidance of unnecessary glucose load, prevention and prompt treatment of hypoglycemia, careful adjustment of nutrition and glucose-containing fluids, and integration of GV with organ dysfunction, vasoactive support, infection status, and PELOD-2 rather than using GV as an isolated treatment target. Prospective studies should test whether standardized monitoring or continuous glucose monitoring can improve recognition of high-risk glycemic patterns without increasing hypoglycemia. 

## 5. Conclusions

In conclusion, early glucose variability during the first 72 h of PICU admission was associated with illness severity and mortality in this single-center retrospective cohort of critically ill children without diabetes. Among the evaluated indices, SD showed the strongest association with mortality in this dataset and remained associated with outcome together with PELOD-2 in an exploratory model. These findings should be considered hypothesis-generating and do not establish causality or prove incremental prognostic utility. Prospective multicenter studies using standardized or continuous glucose monitoring are required to validate the optimal GV metric and to determine whether GV-informed monitoring can improve clinically meaningful outcomes.

## Figures and Tables

**Table 1:** Baseline characteristics of the study population.

Patient Characteristics	Total	Survivors	Non-Survivors	*p*-Value
Participants, *n* (%)	248 (100)	230 (92.7)	18 (7.3)	
Sex, *n* (%)				0.758
Male	143 (57.7%)	132 (57.4%)	11 (61.1%)	
Female	105 (42.3%)	98 (42.6%)	7 (38.9%)	
Age (years), mean ± SD	7.09 ± 5.9	6.89 ± 5.8	9.29 ± 6.7	0.096
BMI (kg/m^2^), mean ± SD	18.3 ± 10.5	18.5 ± 10.8	16.9 ± 3.9	0.535
Comorbidities, *n* (%)	70 (28.2%)	60 (26.1%)	10 (55.6%)	0.007
Enteral nutrition, *n* (%)	184 (75.1%)	174 (76.7%)	10 (55.6%)	0.046
Mechanical ventilation, *n* (%)	187 (75.4%)	169 (73.5%)	18 (100%)	0.012
Vasoactive agents, *n* (%)	162 (65.3%)	146 (63.5%)	16 (88.9%)	0.029
LOS (days), median (IQR)	7.0 (4–15)	7.0 (4–14)	12.0 (4–44)	0.186
Duration of mechanical ventilation (days), mean ± SD	7.89 ± 7.0	7.69 ± 6.8	9.80 ± 8.9	0.269
PELOD-2, median (IQR)	3.0 (1–5)	3.0 (1–5)	12.5 (5–15)	<0.001

BMI, Body Mass Index; LOS, Length of Stay; IQR, Interquartile Range; SD, Standard Deviation.

**Table 2:** Blood glucose measurements during the first 72 h of PICU stay.

Glucose Measurements	Total	Survivors	Non-Survivors	*p*-Value
Participants, *n* (%)	248 (100)	230 (92.7)	18 (7.3)	
Number of glucose measurements, Days 1–3, mean ± SD	15.9 ± 4.0	15.8 ± 4.1	17.5 ± 3.3	0.186
	Glucose concentrations (mg/dL)			
Day 1, average, mean ± SD	133 ± 49 **	132 ± 48 **	149 ± 57	0.140
Day 2, average, mean ± SD	118 ± 27 *	118 ± 27 *	115 ± 21	0.551
Day 3, average, mean ± SD	117 ± 28 *	116 ± 28 *	125 ± 25	0.278
Day 1, max, mean ± SD	158 ± 72 **	157 ± 72 **	178 ± 73	0.255
Day 2, max, mean ± SD	144 ± 51 *	144 ± 52 *	143 ± 40	0.918
Day 3, max, mean ± SD	142 ± 44 *	140 ± 44 *	159 ± 46	0.092
Day 1, min, mean ± SD	110 ± 39 **	110 ± 39 **	103 ± 32	0.458
Day 2, min, mean ± SD	97 ± 23 *	97 ± 23 *	89 ± 21	0.142
Day 3, min, mean ± SD	98 ± 21 *	99 ± 23 *	93 ± 13	0.265

Longitudinal comparisons were performed using the Friedman test for related samples. * *p* < 0.05 for the comparison between Day 1 and either Day 2 or Day 3; ** *p* < 0.05 for both comparisons, Day 1 vs. Day 2 and Day 1 vs. Day 3. Detailed *p*-values are described in the text.

**Table 3:** Comparative results of five glucose variability indices between survivors and non-survivors.

Glucose Variability	Total	Survivors	Non-Survivors	*p*-Value
Participants, *n* (%)	248 (100) *	230 (92.7)	18 (7.3)	
MAG, mean ± SD	13.7 ± 9.36	13.3 ± 8.7	19.4 ± 14.5	0.007
CV, mean ± SD	0.26 ± 0.18	0.25 ± 0.18	0.37 ± 0.24	0.006
SD, mean ± SD	1.48 ± 0.90	1.34 ± 0.80	3.2 ± 1.46	<0.001
GLI, mean ± SD	8.5 ± 15.2	7.1 ± 9.95	23.7 ± 41.3	<0.001
ACACP, mean ± SD	42.1 ± 16.7	41.4 ± 16.2	51.7 ± 21.2	0.012

* GLI was available in 238 patients; all other variables shown in the table were available in 248 patients.

**Table 4:** Correlations of glucose variability indices with glucose measurements, lactate levels, and illness severity during the first 3 days of ICU stay.

Glucose (Serum Levels)	MAG	CV	SD	GLI	ACACP
Mean Day 1	r = 0.48, *p* < 0.001	r = 0.30, *p* < 0.001	r = 0.19, *p* = 0.003	r = 0.19, *p* = 0.007	r = 0.33, *p* < 0.001
Mean Day 2	r = 0.41, *p* < 0.001	r = 0.27, *p* < 0.001	r = 0.15, *p* = 0.017	r = −0.06, *p* = 0.433	r = 0.35, *p* < 0.001
Mean Day 3	r = 0.31, *p* < 0.001	r = 0.24, *p* < 0.001	r = 0.19, *p* = 0.002	r = −0.11, *p* = 0.111	r = 0.19, *p* = 0.002
Highest Day 1	r = 0.55, *p* < 0.001	r = 0.33, *p* < 0.001	r = 0.24, *p* < 0.001	r = 0.21, *p* = 0.003	r = 0.26, *p* < 0.001
Highest Day 2	r = 0.48, *p* < 0.001	r = 0.31, *p* < 0.001	r = 0.19, *p* = 0.003	r = 0.06, *p* = 0.382	r = 0.28, *p* < 0.001
Highest Day 3	r = 0.41, *p* < 0.001	r = 0.31, *p* < 0.001	r = 0.25, *p* < 0.001	r = 0.11, *p* = 0.117	r = 0.25, *p* < 0.001
Lowest Day 1	r = 0.16, *p* = 0.013	r = 0.14, *p* = 0.030	r = 0.01, *p* = 0.873	r = 0.13, *p* = 0.065	r = 0.20, *p* = 0.002
Lowest Day 2	r = 0.09, *p* = 0.13	r = 0.05, *p* = 0.425	r = −0.05, *p* = 0.392	r = 0.03, *p* = 0.699	r = 0.20, *p* = 0.001
Lowest Day 3	r = 0.01, *p* = 0.903	r = 0.04, *p* = 0.545	r = 0.02, *p* = 0.810	r = −0.03, *p* = 0.648	r = 0.07, *p* = 0.268
Lactate Day 1	r = 0.21, *p* = 0.002	r = 0.20, *p* = 0.004	r = −0.10, *p* = 0.133	r = 0.01, *p* = 0.841	r = 0.11, *p* = 0.114
PELOD-2 Day 1	r = 0.13, *p* < 0.05	r = 0.22, *p* < 0.001	r = 0.33, *p* < 0.001	r = 0.23, *p* = 0.001	r = 0.13, *p* < 0.04

**Table 5:** Receiver operating characteristic curve analysis of glucose variability indices and PELOD-2 for mortality prediction.

	Area Under the ROC Curve					
Test Result Variable(s)	*n*	Area	Std. Error ^a^	Asymptotic Sig. ^b^	Asymptotic 95% Confidence Interval	
					Lower Bound	Upper Bound
**CV**	248	0.664	0.074	0.027	0.519	0.809
**SD**	248	0.877	0.046	<0.001	0.786	0.968
**GLI**	238	0.698	0.069	0.004	0.563	0.833
**MAG**	248	0.573	0.086	0.393	0.405	0.742
**ACACP**	248	0.684	0.066	0.005	0.555	0.814
**PELOD-2**	248	0.875	0.046	<0.001	0.785	0.965

^a^ Under the nonparametric assumption. ^b^ Null hypothesis: true area = 0.5.

**Table 6:** Pairwise comparison of the area under the ROC curves between PELOD-2 and SD using DeLong’s non-parametric test.

Pair	AUC PELOD-2	AUC SD	ΔAUC	z	*p*-Value	95% CI for ΔAUC
**PELOD-2 vs. SD**	0.882	0.882	0.002	0.006	0.995	−0.118 to 0.119

## Data Availability

The data supporting the findings of this study are summarized within the article and Supplementary Materials. The underlying anonymized dataset is not publicly available because of institutional, ethical, and patient confidentiality restrictions, but may be made available from the corresponding author upon reasonable request and subject to institutional approval.

## Supplementary Materials

The following supporting information can be downloaded at: https://www.mdpi.com/article/10.3390/nu18142304/s1, Table S1: Intravenous fluids, fluid balance, and enteral nutrition during the first 72 h of PICU stay; Figure S1: GV distribution in different types of IV solutions or their combination administered to patients who survived or died in the PICU; Table S2: Admission laboratory findings; Supplementary ROC coordinate analysis: Youden-derived cut-off values for glucose variability indices and PELOD-2.

## Abbreviations

The following abbreviations are used in this manuscript:

PICU	Pediatric Intensive Care Unit
GV	Glycemic variability
SD	Standard Deviation
CV	Coefficient of Variation
MAG	Mean Absolute Glucose Change
GLI	Glycemic Lability Index
ACACP	Average Consecutive Absolute Change Percentage
BMI	Body Mass Index
PELOD-2	Pediatric Logistic Organ Dysfunction-2
ROC	Receiver Operating Characteristic

## References

[1] Rake A.J., Srinivasan V., Nadkarni V., Kaptan R., Newth C.J.L. (2010). Glucose Variability and Survival in Critically Ill Children: Allostasis or Harm?. Pediatr. Crit. Care Med..

[2] Suh S., Kim J.H. (2015). Glycemic Variability: How Do We Measure It and Why Is It Important?. Diabetes Metab. J..

[3] Du Y., Liu C., Li J., Dang H., Zhou F., Sun Y., Xu F. (2020). Glycemic Variability: An Independent Predictor of Mortality and the Impact of Age in Pediatric Intensive Care Unit. Front. Pediatr..

[4] Dong M., Liu W., Luo Y., Li J., Huang B., Zou Y., Liu F., Zhang G., Chen J., Jiang J. (2022). Glycemic Variability Is Independently Associated with Poor Prognosis in Five Pediatric ICU Centers in Southwest China. Front. Nutr..

[5] Moscardó V., Giménez M., Oliver N., Hill N.R. (2020). Updated Software for Automated Assessment of Glucose Variability and Quality of Glycemic Control in Diabetes. Diabetes Technol. Ther..

[6] Marics G., Lendvai Z., Lódi C., Koncz L., Zakariás D., Schuster G., Mikos B., Hermann C., Szabó A.J., Tóth-Heyn P. (2015). Evaluation of an Open Access Software for Calculating Glucose Variability Parameters of a Continuous Glucose Monitoring System Applied at Pediatric Intensive Care Unit. Biomed. Eng. Online.

[7] Palaiodimou L., Lioutas V.-A., Lambadiari V., Theodorou A., Themistocleous M., Aponte L., Papagiannopoulou G., Foska A., Bakola E., Quispe R. (2021). Glycemic Variability of Acute Stroke Patients and Clinical Outcomes: A Continuous Glucose Monitoring Study. Ther. Adv. Neurol. Disord..

[8] Finfer S., Chittock D.R., Su S.Y.-S., Blair D., Foster D., Dhingra V., Bellomo R., Cook D., Dodek P., NICE-SUGAR Study Investigators (2009). Intensive versus Conventional Glucose Control in Critically Ill Patients. N. Engl. J. Med..

[9] Ali N.A., O’Brien J.M., Dungan K., Phillips G., Marsh C.B., Lemeshow S., Connors A.F., Preiser J.-C. (2008). Glucose Variability and Mortality in Patients with Sepsis. Crit. Care Med..

[10] Krinsley J.S. (2009). Glycemic Variability and Mortality in Critically Ill Patients: The Impact of Diabetes. J. Diabetes Sci. Technol..

[11] Hanna M., Balintescu A., Glassford N., Lipcsey M., Eastwood G., Oldner A., Bellomo R., Mårtensson J. (2021). Glycemic Lability Index and Mortality in Critically Ill Patients-A Multicenter Cohort Study. Acta Anaesthesiol. Scand..

[12] Lu Z., Tao G., Sun X., Zhang Y., Jiang M., Liu Y., Ling M., Zhang J., Xiao W., Hua T. (2022). Association of Blood Glucose Level and Glycemic Variability with Mortality in Sepsis Patients During ICU Hospitalization. Front. Public Health.

[13] Miao G., Lu R., Pipanmekaporn T., Kacha S., Supphapipat A., Phothikun N., Jewprasertpan P., Chittawatanarat K. (2026). Association Between Blood Glucose Variability and Clinical Outcomes in Patients with Sepsis: A Systematic Review and Meta-Analysis. Diabetes Metab. Res. Rev..

[14] Zhou J., Chen Z., Huang H.-N., Ou C.-Q., Li X. (2025). Association between Various Blood Glucose Variability-Related Indicators during Early ICU Admission and 28-Day Mortality in Non-Diabetic Patients with Sepsis. Diabetol. Metab. Syndr..

[15] Yadagudde S.P., Jayasenan J., Srinivasagalu K. (2026). Glycaemic Variability and Its Outcome in Intensive Care Unit Patients with Sepsis. J. West Afr. Coll. Surg..

[16] Wang S., Liu L., Li B., Liu Y., Chai Y. (2026). Glycemic Variability as a Predictor of Mortality in Sepsis Patients With Concurrent Persistent Inflammation, Immunosuppression, and Catabolism Syndrome. Immun. Inflamm. Dis..

[17] Ma X., Zhu S., Sun C., Yu Y., Zhao X., Liu F., Lyu J., Sun S. (2025). From Glycemic Variability to Digital Signal Biomarker: A Prognostic and Precision Medicine Framework for Sepsis-Associated Acute Kidney Injury. Ren. Fail..

[18] Feng Y., Liu L., Ren Y., Li Z., Liu Y., Jiang L., Liu Y., He J., Shao Y. (2026). Association between Glycemic Variability and the Risk of Acute Kidney Injury in Patients with Traumatic Brain Injury: A Retrospective Cohort Study with Independent Cohort Analysis. Front. Neurol..

[19] Huang J., Xu J., Zhai Y., Liu S., Duan P., Liang J., Tian Y., Lu M., Liu X., Yu H. (2026). Relationship of Glycemic Variability with Delirium and Mortality among Critically Ill Elderly Patients with Sepsis: A Retrospective Matched Cohort Study. PLoS ONE.

[20] Zhang H.-J., Shuai W.-L., Wu M.-B., Yan W.-C., Fu Y., Zhang H.-Z. (2025). The Additive Effect of Glycemic Variability and Systolic Blood Pressure Variability on Mortality in Patients with Sepsis. BMC Infect. Dis..

[21] Chen H., Lu Y., Bai S., Li Y., Wang T., Cai L., Yang X., Fang Y., Wan J., Tang Y. (2026). Stress-Hyperglycemia Ratio and Glycemic Variability Predict Severity and Mortality in Sepsis-Associated Acute Respiratory Distress Syndrome. World J. Emerg. Med..

[22] Rodbard D. (2007). Optimizing Display, Analysis, Interpretation and Utility of Self-Monitoring of Blood Glucose (SMBG) Data for Management of Patients with Diabetes. J. Diabetes Sci. Technol..

[23] Siegelaar S.E., Holleman F., Hoekstra J.B.L., DeVries J.H. (2010). Glucose Variability; Does It Matter?. Endocr. Rev..

[24] Rodbard D. (2009). Interpretation of Continuous Glucose Monitoring Data: Glycemic Variability and Quality of Glycemic Control. Diabetes Technol. Ther..

[25] Hermanides J., Vriesendorp T.M., Bosman R.J., Zandstra D.F., Hoekstra J.B., Devries J.H. (2010). Glucose Variability Is Associated with Intensive Care Unit Mortality. Crit. Care Med..

[26] Donati A., Damiani E., Domizi R., Botticelli L., Castagnani R., Gabbanelli V., Nataloni S., Carsetti A., Scorcella C., Adrario E. (2014). Glycaemic Variability, Infections and Mortality in a Medical-Surgical Intensive Care Unit. Crit. Care Resusc..

[27] Sadan O., Feng C., Vidakovic B., Mei Y., Martin K., Samuels O., Hall C.L. (2020). Glucose Variability as Measured by Inter-Measurement Percentage Change Is Predictive of In-Patient Mortality in Aneurysmal Subarachnoid Hemorrhage. Neurocrit. Care.

[28] Kapłan C., Kalemba A., Krok M., Krzych Ł. (2022). Effect of Treatment and Nutrition on Glycemic Variability in Critically Ill Patients. Int. J. Environ. Res. Public Health.

[29] Wang F., Guo Y., Jiao C., Zhao S., Sui L., Mao Z., Lu R., Hou R., Zhu X. (2026). Simultaneous Assessment of Stress Hyperglycemia Ratio and Glucose Variability to Predict All-Cause Mortality in Sepsis Patients across Different Glucose Metabolic States: An Observational Cohort Study with Interpretable Machine Learning Approach. Int. J. Surg..

[30] Wang F., Guo Y., Tang Y., Zhao S., Xuan K., Mao Z., Lu R., Hou R., Zhu X. (2025). Combined Assessment of Stress Hyperglycemia Ratio and Glycemic Variability to Predict All-Cause Mortality in Critically Ill Patients with Atherosclerotic Cardiovascular Diseases across Different Glucose Metabolic States: An Observational Cohort Study with Machine Learning. Cardiovasc. Diabetol..

[31] Chen Y., Yang Z., Liu Y., Gue Y., Zhong Z., Chen T., Wang F., McDowell G., Huang B., Lip G.Y.H. (2024). Prognostic Value of Glycaemic Variability for Mortality in Critically Ill Atrial Fibrillation Patients and Mortality Prediction Model Using Machine Learning. Cardiovasc. Diabetol..

