# Supplementary material for: Early Glucose Variability Is Associated with Mortality in Critically Ill Children: A Retrospective Pediatric Intensive Care Study
Source: Nutrients. 2026 Jul 14;18(14):2304. doi: 10.3390/nu18142304 (PMC13416361; doi:10.3390/nu18142304)
Supplement: Supplementary file 1 [file nutrients-18-02304-s001.zip › nutrients-4392764-supplementary.pdf]

Table S1. Intravenous fluids, fluid balance, and enteral nutrition during the first 72 hours of PICU stay.

Daily recordings (ml, kCal/day)	Total	Survivors	Non-survivors	p-value
Participants, n (%)	248 (100)	230 (92.7)	18 (7.3)	
Total fluids, D1, mean±SD	1225±788 *	1228±809	1185±374	0.881
Total fluids, D2, mean±SD	1474±847 *#	1467±846	1569±1053	0.760
Total fluids, D3, mean±SD	1253±742 #	1267±760	1064±380	0.503
Fluid balance, D1, mean±SD	477±609 *	460±613	710±503	0.279
Fluid balance, D2, mean±SD	433±831 *#	438±782	363±1466	0.819
Fluid balance, D3, mean±SD	72±735 #	56.7±736	291±729	0.422
Enteral nutrition D1, mean±SD	174±154 **	177±166	148±98.2	0.764
Enteral nutrition D2, mean±SD	309±196 *	309±197	307±215	0.987
Enteral nutrition D3, mean±SD	412±242 *	403±219	629±612	0.110

** Day 1 vs. Day 2 or Day 3, # Day 2 vs. Day 3, p<0.001 (k-related samples, Friedman test)*

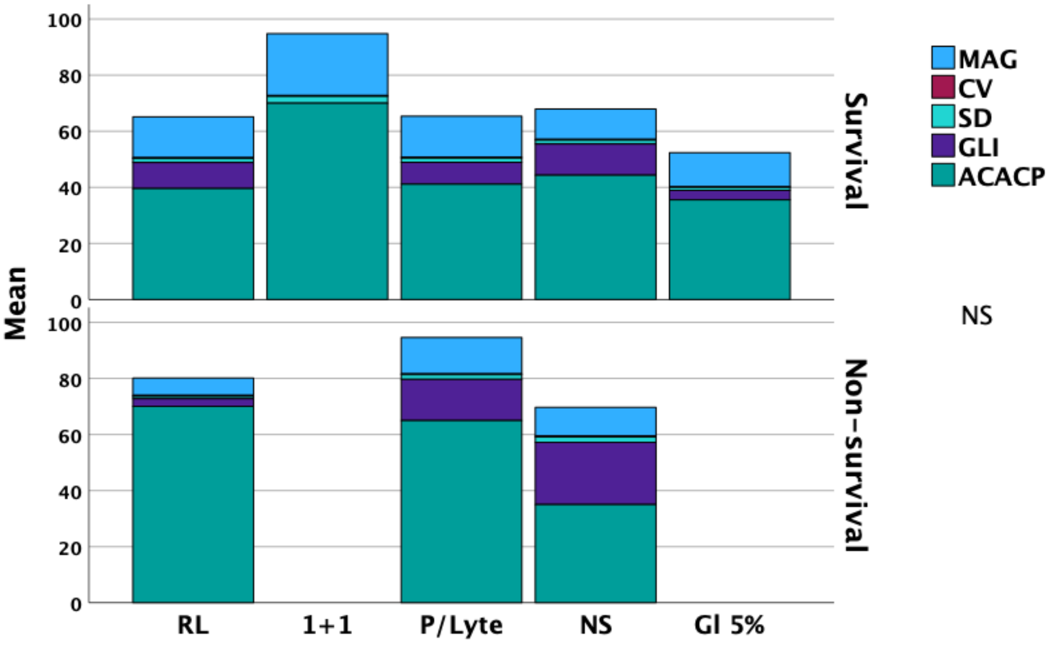

Figure S1. GV distribution in different types of IV solutions or their combination administered to patients who survived or died in the PICU.

Table S2. Admission laboratory findings.

Laboratory assays	Total	Survivors	Non-survivors	p-value
Participants, n (%)	248 (100)	230 (92.7)	18 (7.3)	
K (mEq/L), mean±SD	4.1±0.7	4.1±0.7	3.8±0.4	0.174
Na (mEq/L), mean±SD	136.8±5.3	136.6±5.4	139.0±3.1	0.254
Urea (mg/dl), mean±SD	25.4±15.6	25.1±15.7	30.5±15.1	0.377
Creatinine (mg/dl), mean±SD	0.56±0.22	0.56±0.23	0.51±0.21	0.595
FiO_2, fraction, mean±SD	0.69±0.25	0.71±0.26	0.43±0.1	0.774
pH, mean±SD	7.348±0.11	7.347±0.12	7.330±0.05	0.957
pO_2 (mmHg), mean±SD	149±64	150±64	118±57	0.193
pCO_2 (mmHg), mean±SD	43.1±30	41.5±23	67.1±82	0.031
HCO_3 (mEq/L), mean±SD	23.6±29	23.8±30	19.9±3.3	0.737
BE (mEq/L), mean±SD	-4.1±5.2	-3.9±5.3	-5.3±3.5	0.390
Lactate (mg/dl), mean±SD	1.9±2.7	2.0±2.7	1.5±2.0	0.609

Supplementary ROC coordinate analysis

Youden-derived cut-off values for glucose variability indices and PELOD-2.

Among glucose variability indices, SD had the highest Youden's index, with an optimal cut-off of ≥2.68, sensitivity of 76.5%, specificity of 91.7%, and Youden's index of 0.682. The corresponding cut-offs were ≥0.338 for CV, ≥18.90 for GLI, ≥14.03 for MAG, and ≥35.4 for ACACP. For PeLOD-2, the optimal Youden-derived cut-off was ≥4.5, with sensitivity of 88.2%, specificity of 70.2%, and Youden's index of 0.584. A higher PELOD-2 threshold of ≥11.5 provided markedly higher specificity (98.9%) but lower sensitivity (58.8%), with a similar Youden's index of 0.577.
